# Dementia With Lewy Bodies: A Review of Disease-Modifying Therapies for α-Synucleinopathies

**DOI:** 10.1192/bjo.2025.10792

**Published:** 2025-06-20

**Authors:** Arzoo Dar, Sharmi Bhattacharyya

**Affiliations:** 1 Hull York Medical School, York, United Kingdom; 2 University of Chester, Chester, United Kingdom

## Abstract

**Aims:** The global demographic transition has led to a growing ageing population. Neurodegenerative disease prevalence is high in the elderly, as aged brains accumulate molecular and cellular damage. Protein misfolding and aggregation are pathological processes underlying neurodegeneration, as seen in dementia. This coupled with oxidative stress, disrupted ubiquitin-proteosome system and neuroinflammation may contribute to neuronal damage/death. Lewy Body dementia (LBD) collectively refers to Parkinson’s disease (PD), Parkinson-disease dementia and dementia with Lewy bodies (DLB). It is characterised by Lewy Body (LB) deposits in the central nervous system, predominantly compromised of misfolded α-synuclein protein. Disease-modifying therapy (DMT) targets underlying protein misfolding and promotes LB clearance.

To review international trials on DMT safety, tolerability and efficacy in DLB.

To consider the molecular basis for protein misfolding and DMT in DLB.

**Methods:** Two international registries (ClinicalTrials.gov; EU clinical trials register) were consulted to identify agents being tested in completed, ongoing and recruiting trials. Both databases were searched by disease “Lewy body dementia” or “LBD” or “alpha-synuclein pathology” and study type “interventional” for phase I–III trials. Trials solely investigating diagnostic biomarkers, approved symptomatic DLB treatment or PD without dementia symptoms were excluded from final analysis.

**Results:** 11 trials were found studying DMTs for DLB. 10 out of the 11 trials were in phase II and one in phase I. The main agents investigated were tyrosine-kinase inhibitors (TKIs), phosphodiesterase-9 inhibitors, β-adrenergic agonists, and β GCase chaperones. Primary and secondary outcome measures were “safety/tolerability of agent”, “changes in cognitive function” or “changes in serum/CSF α synuclein levels”. Results from a phase I trial demonstrated a 3.85 and 3.5-point increase in mini-mental state examination scores at 6 months with nilotinib doses 300 mg and 150 mg, respectively. Contrastingly, only slight reduction in CSF/plasma α-synuclein was observed. Phase II trials for bosutinib versus placebo showed no significant difference in cognitive function. Trials for ambroxol, a β-GCase enzyme enhancing inhibitory chaperone, remain in recruiting stages but have proven drug tolerability due to fewer side effects. Data on CSF biomarker changes in DLB was unavailable.

**Conclusion:** Current understanding of protein misfolding in α-synucleinopathies suggests that a single DMT may be insufficient in mixed pathology. Cocktail therapy targeting various misfolded proteins may be necessary for a cure. DMTs have limited use as most patients are diagnosed with advanced DLB. Sensitive diagnostic biomarkers with high specificity are required for accurate DLB diagnosis in the prodromal phase, a critical window for protein misfolding reversal with DMT.

